# How to publish a lot—The sequel to writing

**DOI:** 10.1002/lio2.1142

**Published:** 2023-08-25

**Authors:** Romaine F. Johnson

**Affiliations:** ^1^ Department of Otolaryngology‐Head and Neck Surgery UT Southwestern Medical Center Dallas Texas USA

**Keywords:** academic writing, publication strategies, writing productivity

In the world of academia, the adage “publish or perish” resonates deeply. Building on the foundational insights of Dr. Paul J. Silvia's “How to Write a Lot,^1^” this essay embarks on the next phase of the academic journey: how to “publish” a lot. While the act of writing is foundational, the true testament of its value crystallizes in its publication. To draw an analogy, just as a tree falling in a forest might not make a sound if no one is there to hear it, research that remains unpublished might not make an impact if it remains unread. In the academic realm, publications serve as both a badge of honor and the primary currency. They amplify your reputation, paving the way for lectures, keynotes, committee chairs, and academic promotions.

However, the path to publication is covered with challenges. As an Editor‐in‐Chief, I reject 75% of the articles under my purview. Some journals wield an even stricter sieve, declining as many as 90% of submissions. The sting of rejection, especially after months of dedicated effort, is a sentiment many of us are familiar with. The prolonged waiting period and potential rejections mean that one might invest a year or even longer on a project without seeing a tangible outcome.

Before we delve into the strategies that can bolster your publishing success, we must address a foundational question: what should one write about? While conventional wisdom often nudges young researchers toward finding a niche, I advocate for a more intrinsic approach: write about what ignites your passion. Knowledge gaps are omnipresent, waiting for the right mind to bridge them. This essay, the first in a four‐part series, aims to guide you through the labyrinthine publishing world, ensuring your hard work gets its deserved spotlight.

A cornerstone of successful publishing is ensuring your research is anchored in a clear scientific rationale. This clarity should permeate right from the introduction. Here's a template to guide your narrative:

1. Define a problem.
2. Provide evidence that it is, indeed, a problem.
3. Highlight the existing knowledge gap.
4. Demonstrate how your paper will bridge this gap.

Consider this illustrative example: “Children with tracheostomies are among the most complex patients in the healthcare system, often requiring 24‐h nursing care. While some eventually transition to a life without tracheostomy, many carry it into adulthood. Their nursing needs evolve, yet there's a prominent absence of evidence‐based guidelines for pediatric‐to‐adult transition care for these patients. The Pediatric Tracheostomy Clinic, a multidisciplinary clinic, has previously published quality improvement initiatives on tracheostomy care. It is therefore uniquely poised to analyze this transition. This study seeks to examine an adult transition program for pediatric tracheostomy patients, hypothesizing that a multidisciplinary approach will smoothen this transition.”

Many manuscripts falter by not adhering to this structured approach, leading to premature rejection. The essence and significance of your research should be clearly articulated. As the African American vernacular wisely advises, “Make it plain” or “Put it where the goats can get it.”

The methods section often becomes the Achilles' heel for many papers. Think of it as a cookbook that lists ingredients but omits the baking instructions. Your research methods should be a beacon, guiding replication efforts. Eschew vague statements about data collection or analysis techniques. Instead, detail every step, ensuring another researcher can walk in your shoes without stumbling.

For instance, a statement like “We performed a retrospective study of tracheostomy patients, collecting demographics and surgical outcomes, and used statistical tests to estimate postoperative complications” provides a glimpse but lacks depth. Contrast this with a more detailed account: “This study, approved by the University's IRB (#IRB number), was a retrospective chart review of tracheostomy patients ages 18–99 years old. Using the electronic medical record's search engine (Epic Hyperspace), we identified patients who underwent tracheostomy between January 1, 2020, and December 31, 2021. CPT codes 31600‐01 were used to identify patients. We collected the following variables—age in years, gender (male or female), race (Black, White, Other), and the principal reason for tracheostomy (airway obstruction or respiratory failure).”

The difference is noticeable. Detailed descriptions allow reviewers and editors to trace your steps, setting clear expectations for the results. Catching the reader off‐guard with unexpected findings can muddle the narrative, and a perplexed reviewer often leans toward rejection.

Results should be woven seamlessly into the narrative, not relegated to tables. A results section that is peppered with “see Table X” will quickly find itself in the rejection binder. Begin by painting a picture of the study population, then segue into primary and secondary findings, and round off with any sensitivity analyses or tests for interactions. This structured narrative ensures a holistic portrayal.

In the discussion section, it is paramount to nestle your findings within the broader research landscape, juxtaposing them against similar studies and ruminating on their *generalizability*. Equally vital is a candid introspection into the study's limitations. Instead of skimming the surface (i.e., “this was a retrospective study”), dive deep into specifics. A retrospective study, for example, has many limitations. Try to discuss which one is most pertinent to your study, whether it be measurement sensitivity, lost to follow up, or lack of control over confounding variables.

Writing clarity is nonnegotiable. While many journals champion inclusivity, the lingua franca often remains English. Thus, ensuring readability is pivotal. While professional translation services might strain academic budgets, online grammar tools can be a worthy substitute. As for the burgeoning realm of large‐language model artificial intelligence, we are still charting its potential. While these tools can sharpen the writing edge, they can occasionally introduce nuances that deviate from the intended message as well as introduce false information. Leveraging AI to identify gaps in your narrative might be more judicious than letting it author the entire manuscript. After all, every word bears your signature.

One of my most valuable tools in the writing journey has been references. The EQUATOR network^2^, with its exhaustive checklist for reporting a myriad of biomedical research, has been invaluable. The STROBE guidelines^3^, given their focus on observational research—a mainstay in surgical specialties like otolaryngology—find frequent use in my work. While these guidelines need not be adhered to religiously, juxtaposing your manuscript against them can elevate its quality.

Another gem is “How to Report Statistics in Medicine.^4^” Each chapter, dedicated to a specific study type or statistical test, is a guiding light. For instance, if you are utilizing survival analysis, the book's recommendations can make your narrative clear and concise. Following the template will also help you identify gaps in your methodology and reporting giving you a “heads‐up” for major flaws that will lead to rejection. The authors' simple writing style demystifies statistics, making it accessible to anyone with a foundational biomedical background.

Lastly, the AMA Manual of Style^5^ is indispensable for those serious about publishing. If you are aiming for a narrative that is “by the book,” this is the tome to consult. While an online version is available, I find solace in the tactile experience of flipping through its pages, both at home and in my office. The disclosures, statistical reporting, and inclusive language sections are particularly enlightening.

In wrapping up this first installment, I hope to have illuminated the path from writing to publishing. The journey, while challenging, is immensely rewarding. As we progress in this series, we will delve deeper into the nuances of publishing, from selecting the right journal to navigating the peer review process and beyond. Remember, the essence lies in defining the knowledge gap, detailing your methods with precision, and discussing your findings in their entirety, including potential limitations. Your submissions are eagerly awaited. Here is to your publishing success!

## CONFLICT OF INTEREST STATEMENT

The author has no disclosures or conflicts of interest.
